# The Pretreatment Neutrophil/Lymphocyte Ratio Is Associated with All-Cause Mortality in Black and White Patients with Non-metastatic Breast Cancer

**DOI:** 10.3389/fonc.2016.00081

**Published:** 2016-03-31

**Authors:** Joseph Rimando, Jeff Campbell, Jae Hee Kim, Shou-Ching Tang, Sangmi Kim

**Affiliations:** ^1^Section of Hematology/Oncology, Department of Medicine, Medical College of Georgia at Georgia Regents University, Augusta, GA, USA; ^2^Georgia Regents University Cancer Center, Augusta, GA, USA

**Keywords:** breast cancer, neutrophil/lymphocyte ratio, Black patients, White patients, all-cause mortality

## Abstract

The pretreatment neutrophil/lymphocyte ratio (NLR), derived from differential white blood cell counts, has been previously associated with poor prognosis in breast cancer. Little data exist, however, concerning this association in Black patients, who are known to have lower neutrophil counts than other racial groups. We conducted a retrospective cohort study of 236 Black and 225 non-Hispanic White breast cancer patients treated at a single institution. Neutrophil and lymphocyte counts were obtained from electronic medical records. Univariate and multivariate Cox regression models were used to determine hazard ratios (HRs) and 95% confidence intervals (95% CIs) of all-cause mortality and breast cancer-specific mortality in relation to pretreatment NLR. Overall, there were no associations between an elevated pretreatment NLR (NLR ≥3.7) and all-cause or breast cancer-specific mortality. Among patients without metastasis at the time of diagnosis, an elevated pretreatment NLR was independently associated with all-cause mortality, with a multivariable HR of 2.31 (95% CI: 1.10–4.86). Black patients had significantly lower NLR values than White patients, but there was no evidence suggesting racial heterogeneity of the prognostic utility of NLR. Pretreatment NLR was an independent predictor of all-cause mortality but not breast cancer-specific mortality in non-metastatic breast cancer patients.

## Introduction

Breast cancer is the most common cancer in women and is the second leading cause of cancer death in women in the United States (1). Tumor stage, grade, and tissue markers, such as hormone receptor status and HER2 overexpression, are well-known independent prognostic indicators in breast cancer (2–5). In recent years, increasing data suggest that tumor infiltrating lymphocytes (TILs) may also have prognostic value in breast cancer (6, 7). This interaction between the tumor and immune system may also extend beyond the local tissue environment. Indeed, a systemic inflammatory response, determined by elevated circulating levels of C-reactive protein and interleukin-6, has been associated with poor overall and disease-specific survival among breast cancer patients (8, 9).

The pretreatment neutrophil/lymphocyte ratio (NLR), derived from differential white blood cell (WBC) counts, is another parameter of systemic inflammation and host immune reaction, which is routinely available in the clinical setting. A few studies have reported that an elevated pretreatment NLR is associated with an increased risk of relapse and worse survival among breast cancer patients (10–19). The pretreatment NLR has been associated with worse prognosis in a variety of different cancers, including colorectal cancer (20), non-small cell lung cancer (21, 22), renal cell carcinoma (23, 24), gastric cancer (25, 26), nasopharyngeal carcinoma (27, 28), hepatocellular carcinoma (29), and esophageal cancer (30). Additionally, Chua et al. reported that midtreatment NLR (measured after one cycle of chemotherapy) was associated with improved progression-free survival (*p* = 0.012) in colorectal cancer (31).

Blacks are generally known to have lower neutrophil counts and similar lymphocyte counts compared to their White counterparts (32). However, little data exist concerning the association between the pretreatment NLR and breast cancer prognosis in Black patients. We, therefore, examined whether an elevated pretreatment NLR is associated with all-cause and breast cancer-specific mortality in Black and White patients treated at a single institution.

## Materials and Methods

### Study Design and Data Source

Our study was a retrospective review of medical charts of breast cancer patients registered in the Tumor Registry at the Georgia Regents University (GRU) Cancer Center. Out of 1,208 patients registered between April 2001 and August 2013, we identified 464 patients who had differential WBC counts determined prior to initial treatment and excluded 3 patients who were members of racial/ethnic groups other than Black or non-Hispanic White. The total WBC count and the percentage of each neutrophil and lymphocyte were extracted from electronic medical records (EMR) to estimate the absolute neutrophil and lymphocyte counts. The NLR was then calculated by dividing the estimated neutrophil count by the estimated lymphocyte count. Also extracted from the EMR was information on a patient’s height and weight at the time of diagnosis or at a proximate time possible to compute body mass index (BMI, kilogram per square meter).

Other major information used in this study was obtained from the hospital tumor registry, which abstracts and codes the data on all patients diagnosed and/or treated for cancer at the GRU Hospital following the state cancer registry standards. This included information on patient characteristics (age at diagnosis, race, primary payer at diagnosis, and tobacco use history), tumor (date of diagnosis, stage at diagnosis, grade, and hormone receptor status), treatment course (surgery, radiation, chemotherapy, and hormone therapy), and follow-up data (date of last contact, date and type of recurrence, and vital status). EMR were further searched among patients expired by the follow-up end date in order to determine if death was attributed to breast cancer. Death was considered a result of breast cancer if (1) “a patient was never been free of breast cancer since diagnosis because of already metastasized diseases and/or treatment failure/refusal” or “a patient was disease-free after initial treatment but had a record of disease recurrence” and (2) medical records within 3 months from the expiration date indicate at least one of the following: widespread metastasis into multiple organs; malignant pleural effusion; significant disease progression and functional decline; or referral to hospice care. The study was approved by the Institutional Review Board of GRU. The informed consent process and the documentation of consent was waived because the study was a retrospective chart review that involved no greater than minimal risk and did not collect protected health information.

### Statistical Analysis

Our primary endpoint was overall survival. Overall survival was calculated from the date of diagnosis to the date of death from any causes (censored) or last follow-up (right-censored). A best cutoff point for NLR was established by examining hazard ratios (HRs), SEs, and *p*-values for various cutoff points obtained from a univariate Cox regression (log-rank test) comparing survival curves of individuals above and below each cutoff point (33, 34). Multivariate Cox regression models were used to determine adjusted HRs after controlling for age, stage, grade, and hormone receptor status (adjustment model 1); adjustment model 1 plus smoking and BMI (adjustment model 2); and all adjustment 1 and 2 factors plus payer type, receipt of chemotherapy, and receipt of radiation therapy (adjustment model 3). A test for linear trend between stage and NLR was conducted by using univariate linear regression. In addition to an analysis, including all patients, we conducted subgroup analyses by race and metastasis status. All analyses were performed in R 3.2.

## Results

This study included 461 (236 Black and 225 White) patients with breast cancer (Table 1). Overall, 122 (65 Black and 57 White) patients died during a median follow-up of 5.1 years [interquartile range (IQR): 5.4]. The median age at diagnosis was 57.8 years (IQR: 19). The median BMI was 29.5 kg/m^2^ (IQR: 9.4), and 20% of patients were current smokers. A majority of patients (90.1%) had some type of health insurance at the time of diagnosis, and 93% had a surgery as a part of the treatment compared with 51 and 46% having radiation and chemotherapy, respectively.

**Table 1:** **Patient and clinical characteristics and their relationship with overall survival in a hospital-based retrospective cohort study (2001–2013)**.

	No. (%)	Unadjusted HR (95% CI)
**Age**		
<50	129 (28.0%)	1.22 (0.79, 1.88)
50–64	186 (40.3%)	1. (Ref.)
≥65	146 (31.7%)	1.29 (0.84, 1.99)
Missing	0	–
**Race**		
Black	236 (51.2%)	1.08 (0.76, 1.54)
Non-Hispanic White	225 (48.8%)	1. (Ref.)
**BMI**		
<25	108 (25.2%)	1. (Ref.)
25–29.9	121 (28.2%)	0.53 (0.31, 0.92)
30–34.9	102 (23.8%)	0.91 (0.54, 1.52)
≥35	98 (22.8%)	0.87 (0.51, 1.48)
Missing	32	–
**Smoking**		
Never	254 (61.1%)	1. (Ref.)
Former	78 (18.8%)	1.14 (0.69, 1.90)
Current	84 (20.2%)	1.42 (0.90, 2.23)
Missing	45	–
**Type of medical insurance**		
Private/Medicare/uniformed	337 (74.2%)	1. (Ref.)
Medicaid	73 (16.1%)	2.22 (1.46, 3.38)
None	44 (9.7%)	1.64 (0.95, 2.83)
Missing	7	–
**Stage**		
0 and I	213 (52.4%)	1. (Ref.)
II	114 (28.1%)	2.54 (1.44, 4.50)
III	62 (15.3%)	4.68 (2.61, 8.38)
IV	17 (4.2%)	12.60 (6.23, 25.30)
Missing	7	0
**Grade**		
I	84 (21.2%)	1. (Ref.)
II	115 (29.0%)	1.67 (0.85, 3.27)
III	193 (48.7%)	2.18 (1.18, 4.04)
IV	4 (1.0%)	
Missing	65	–
**Hormone receptor status**		
ER+, PR+	244 (61.9%)	1. (Ref.)
ER+, PR−	46 (11.7%)	
ER−, PR+	9 (2.328%)	
ER−, PR−	95 (24.1%)	2.18 (1.45, 3.28)
Missing	67	–
**Type of therapy**		
Surgery	427/461 (92.6)	0.13 (0.08, 0.20)
Radiation therapy	232/452 (51.3)	0.63 (0.44, 0.91)
Chemotherapy	210/458 (45.9)	1.77 (1.22, 2.56)
Systemic hormone therapy	234/445 (52.6)	0.54 (0.37, 0.78)
**Median (interquartile range)**		
Total WBC	6.7 ± 2.9	0.98 (0.91, 1.05)
Neutrophil count	4.1 ± 2.2	1.02 (0.94, 1.11)
Lymphocyte count	2.0 ± 0.8	0.74 (0.57, 0.95)

Approximately half of the patients had *in situ* (*N* = 81 or 19%) or stage I (*N* = 149 or 34%) tumors, followed by 28% of patients with stage II, 15% with stage III, and 4% with stage IV. The proportion of patients with grade I, II, III, and IV tumors was 21, 29, 49, and 1%, respectively. The most frequent tumor subtype was the subtype positive for both the estrogen receptor (ER) and progesterone receptor (PR) (62%). The median counts of WBCs, neutrophils, and lymphocytes were 6.7, 4.1, and 2, respectively.

As expected, the HRs of all-cause mortality increased in relation to advanced tumor stage [HR for stage IV versus stage 0 or I: 12.60, 95% confidence interval (95% CI): 6.23–25.30], higher grade (HR for grade III/IV versus grade I: 2.18, 95% CI: 1.18–4.04), and negative hormone receptor status (HR for ER− and PR− versus ER+ or PR+: 2.18, 95% CI: 1.45–3.28). Having Medicaid as a primary payer source and receiving chemotherapy were also associated with elevated risk of all-cause mortality in univariate analyses, but other patient factors, such as age, race, BMI, and smoking history, were not associated with overall survival. No relationship was observed with WBC, neutrophil, or lymphocyte counts.

**Figure 1:**
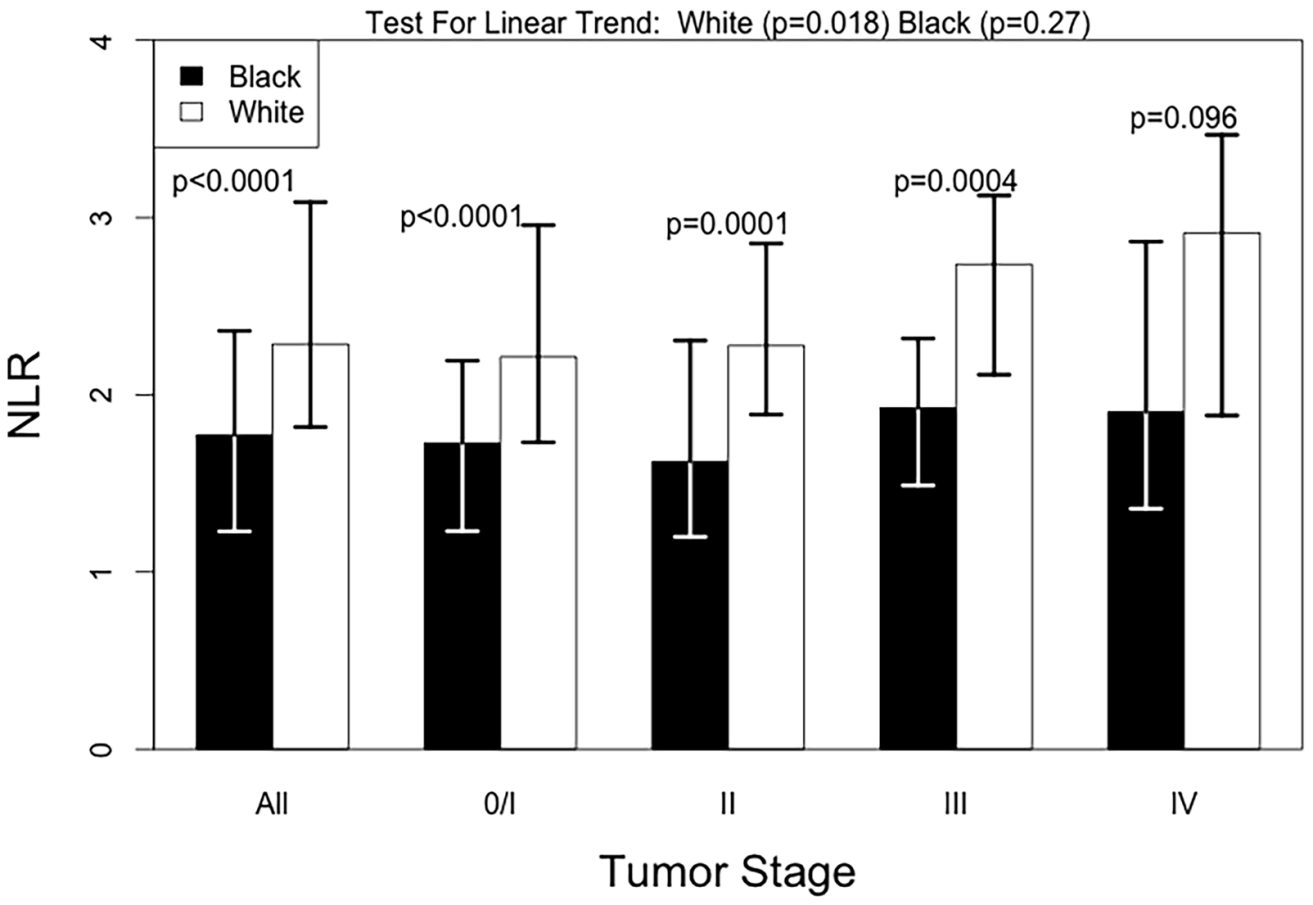
**Median NLR levels by tumor stage, according to race**. Test for linear trend was done with univariate linear regression and tests by stage were done using the Mann–Whitney test.

**Figure 2:**
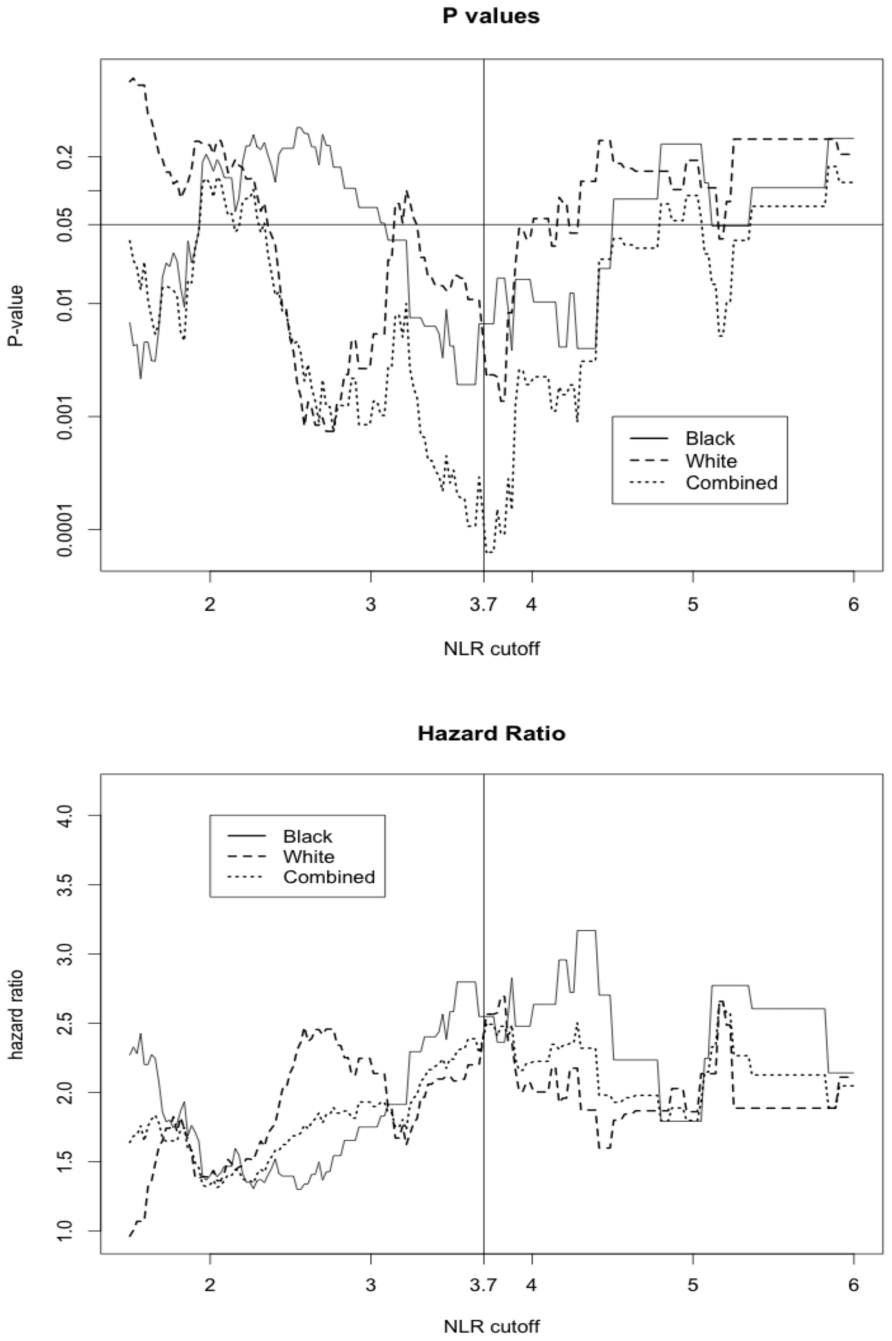
**The hazard ratio and *p*-value for all-cause mortality in patients above or equal versus below the corresponding NLR cutoff displayed on *x*-axis**.

Black patients had lower NLR values compared with White patients, with a median NLR (IQR) of 1.77 (1.14) for Black patients and 2.28 (1.25) for White patients. This racial difference in NLR remained even when stratified by tumor stage [all *p*-values <0.001 except for the stage IV (*p* = 0.096)] (Figure 1). Interestingly, a positive association between tumor stage and the NLR value was evident only among White patients (*p*-value for a linear trend = 0.02) and was not observed in Black patients (*p*-value for a linear trend = 0.27). Due to differences in NLR between Black and White patients, we calculated an optimal NLR cutoff point that would significantly predict all-cause mortality in each racial group and in the combined patient population (Figure 2; Table 2). A high HR with the lowest *p*-value was observed at the NLR cutoff value of 3.6 among Black patients and at 3.8 among White patients. However, there was only little difference in the HRs and the associated *p*-values in a range of NLR cutoff values between 3.6 and 3.8 in both racial groups. We thus used an overall optimal cutoff point of 3.7 in subsequent analyses.

**Table 2:** **Race-specific and overall HRs and *p*-values for all-cause mortality across different NLR cutoff points**.

NLR cutoff	Black			White			All		
	HR	SE	*p*	HR	SE	*p*	HR	SE	*p*
2.0	1.398	0.250	0.180	1.392	0.290	0.254	1.333	0.183	0.116
2.1	1.467	0.253	0.130	1.420	0.285	0.219	1.367	0.182	0.085
2.2	1.500	0.257	0.115	1.471	0.275	0.160	1.416	0.181	0.055
2.3	1.374	0.265	0.231	1.650	0.272	0.066	1.446	0.182	0.043
2.4	1.522	0.269	0.119	1.777	0.270	0.033	1.583	0.182	0.012
2.5	1.397	0.283	0.237	2.137	0.271	0.005	1.666	0.184	0.005
2.6	1.340	0.295	0.320	2.368	0.269	0.001	1.737	0.185	0.003
2.7	1.366	0.310	0.315	2.416	0.267	0.001	1.780	0.188	0.002
2.8	1.544	0.310	0.161	2.338	0.266	0.001	1.850	0.191	0.001
2.9	1.654	0.310	0.105	2.126	0.267	0.005	1.815	0.194	0.002
3.0	1.752	0.310	0.071	2.246	0.269	0.003	1.931	0.197	0.001
3.1	1.914	0.310	0.036	1.886	0.282	0.024	1.854	0.206	0.003
3.2	1.914	0.310	0.036	1.759	0.290	0.052	1.804	0.210	0.005
3.3	2.294	0.311	0.007	1.814	0.303	0.049	1.987	0.216	0.001
3.4	2.439	0.321	0.005	2.097	0.303	0.014	2.204	0.218	>0.001
3.5	2.583	0.332	0.004	2.118	0.303	0.013	2.245	0.221	>0.001
3.6	2.797	0.332	0.002	2.096	0.309	0.017	2.314	0.225	>0.001
3.7	2.548	0.344	0.007	2.369	0.309	0.005	2.381	0.228	>0.001
3.8	2.363	0.360	0.017	2.693	0.309	0.001	2.478	0.232	>0.001
3.9	2.478	0.378	0.016	2.162	0.337	0.022	2.238	0.250	0.001
4.0	2.478	0.378	0.016	2.072	0.350	0.037	2.210	0.256	0.002
4.1	2.636	0.378	0.010	2.004	0.365	0.057	2.225	0.262	0.002
4.2	2.957	0.378	0.004	1.959	0.383	0.079	2.338	0.269	0.002
4.3	3.168	0.401	0.004	1.873	0.405	0.121	2.320	0.284	0.003
4.4	3.168	0.401	0.004	1.873	0.405	0.121	2.320	0.284	0.003
4.5	2.703	0.429	0.020	1.600	0.433	0.278	1.981	0.304	0.020

We further explored other patient parameters associated with the pretreatment NLR value (Table 3). Patients in the high NLR group defined as NLR ≥3.7 had higher WBC and neutrophil counts but lower lymphocyte counts compared to those in the low NLR group (NLR <3.7) (all *p*-values <0.001). There were more stage IV (17 versus 8%) and high-grade tumors (67 versus 47%) in the high NLR group than in the low NLR group, but the differences were not statistically significant. Other patient parameters, such as age, BMI, and smoking status, were also not significantly associated with the NLR groups. Using 3.7 as a cutoff point to define the high NLR group, there were fewer Black patients in the high NLR group compared with White patients, but the difference was only borderline significant (39 versus 61%, *p* = 0.07).

**Table 3:** **Comparison of host and tumor characteristics between patients with high NLR (≥3.7) versus low NLR (<3.7) in a hospital-based retrospective cohort study (2001–2013)**.

	NLR **<**3.7	NLR **≥**3.7	*p*-Value
Total no.	409	52	
**Median ± IQR**
White blood cells	6.60 ± 2.70	7.75 ± 3.42	<0.0001
Neutrophils	3.78 ± 2.06	5.82 ± 2.82	<0.0001
Lymphocytes	2.08 ± 0.80	1.22 ± 0.666	<0.0001
Monocytes	0.47 ± 0.23	0.48 ± 0.32	0.75
Age	57.60 ± 19.40	57.40 ± 15.30	0.819
BMI	29.70 ± 9.49	27.5 ± 9.72	0.819
**No. (%)**
**Race**			**0.071**
Black	216 (52.8)	20 (38.5)	
White	193 (47.2)	32 (61.5)	
**Stage**			**0.198**
I	192 (50.7)	20 (41.7)	
II	104 (27.4)	12 (25.0)	
III	53 (14.0)	8 (16.7)	
IV	30 (7.9)	8 (16.7)	
**Grade**			**0.1**
I	78 (22.3)	6 (14.3)	
II	107 (30.6)	8 (19.0)	
III/IV	165 (47.1)	28 (66.7)	
**Smoking**			**0.85**
Never	227 (61.5)	27 (57.4)	
Former	68 (18.4)	10 (21.3)	
Current	74 (20.1)	10 (21.3)	
**Payer**			**0.833**
Private/Medicare/uniformed	301 (74.5)	36 (72.0)	
Medicaid/none	103 (25.5)	14 (28.0)	

*p-Values were obtained using Pearson’s chi-squared tests for categorical variables with simulated p-values used when necessary and the Mann–Whitney test for continuous variables*.

**Figure 3:**
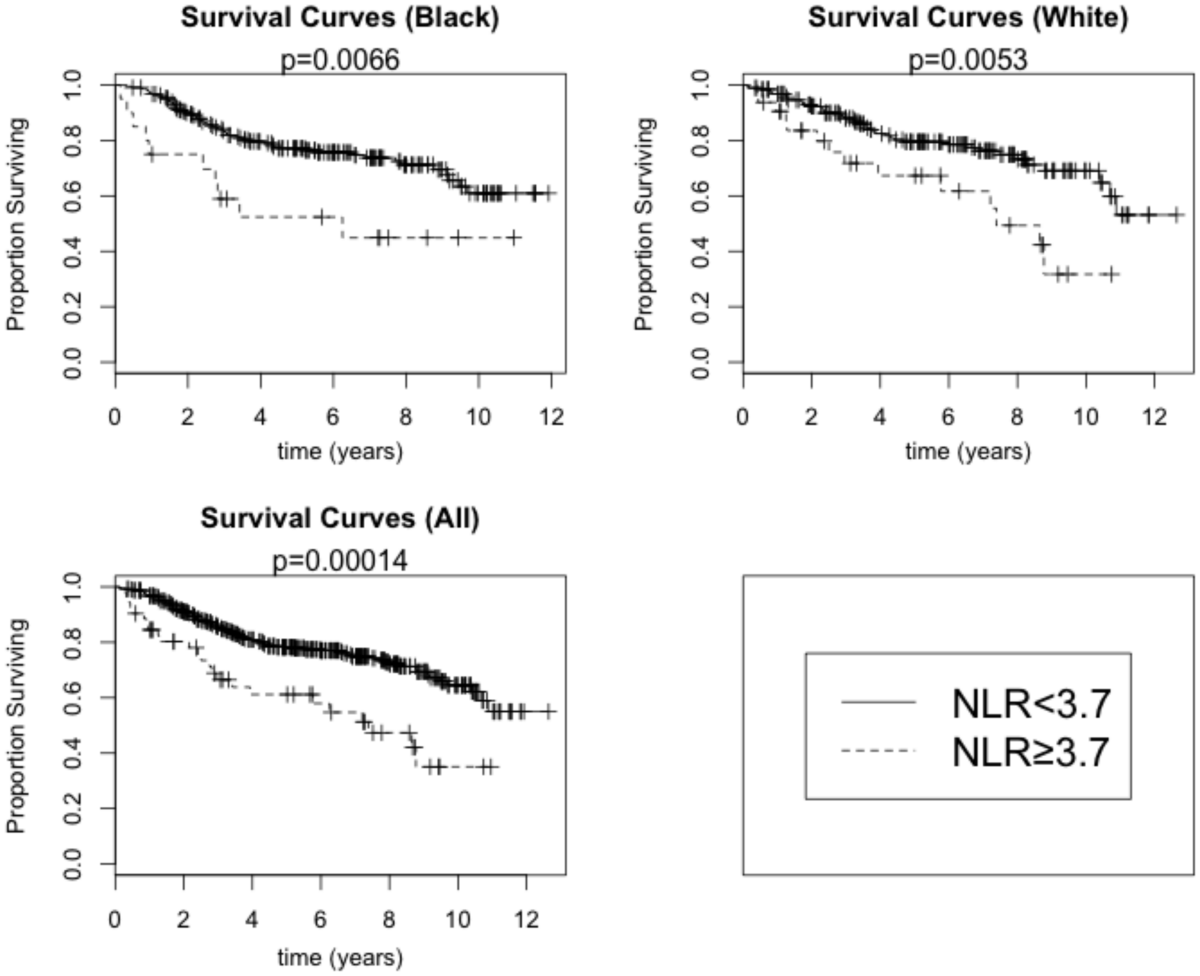
**Overall survival by high NLR (≥3.7) versus low NLR (<3.7) among Black and White breast cancer patients**. *p*-Values are for the log-rank test.

A total of 11% of breast cancer patients (8.5% of Black and 13.8% of White) had a pretreatment value of NLR ≥3.7 and was defined to have an elevated pretreatment NLR. In the univariate, unadjusted analysis, high NLR was associated with increased all-cause mortality (HR = 2.38, 95% CI: 1.52–3.72; *p* < 0.001 by the log-rank test) (Table 4; Figure 3). The univariate HR associated with the high NLR was similar between Black (HR = 2.55, 95% CI: 1.30–5.00) and White (HR = 2.37, 95% CI: 1.29–4.34) patients. After adjustment for age, stage, grade, and combined ER/PR receptor status, however, the high NLR was no longer significantly associated with all-cause mortality (HR = 1.47, 95% CI: 0.80–2.70).

**Table 4:** **All-cause mortality in relation to high NLR (≥3.7) versus low NLR (<3.7)**.

	Black			White			Combined		
	Deaths	HR	95% CI	Deaths	HR	95% CI	Deaths	HR	95% CI
**All stages**									
Unadjusted	65	2.55	(1.30, 5.00)	57	2.37	(1.29, 4.34)	122	2.38	(1.52, 3.72)
Adjustment model 1	44	1.86	(0.78, 4.46)	32	1.04	(0.39, 2.76)	76	1.47	(0.80, 2.70)
Adjustment model 2	35	1.82	(0.66, 5.05)	25	1.76	(0.56, 5.57)	60	1.52	(0.79, 2.91)
Adjustment model 3	35	1.3	(0.43, 3.91)	25	1.49	(0.44, 5.07)	60	1.47	(0.73, 2.96)
**Excluding stage IV**									
Unadjusted	40	2.78	(1.16, 6.64)	35	2.28	(1.03, 5.03)	75	2.43	(1.36, 4.35)
Adjustment model 1	34	2.53	(0.97, 6.62)	23	1.68	(0.51, 5.53)	57	2.12	(1.05, 4.29)
Adjustment model 2	25	3.68	(1.04, 13.00)	18	3.17	(0.82, 12.3)	43	2.44	(1.12, 5.31)
Adjustment model 3	25	2.88	(0.72, 11.60)	18	2.86	(0.69, 11.8)	43	2.27	(1.02, 5.08)

*Adjustment model 1 controlled for stage (0/I, II, III, and IV), grade (I, II, and III/IV), hormone receptor status (ER+ or PR+, ER−, and PR−), and age (continuous). In addition to the variables in adjustment model 1, adjustment model 2 controlled for smoking (former and current versus never) and BMI (<25, 25–2.9, 30–34.9, and 35+). In addition to the variables in adjustment model 2, adjustment model 3 controlled for payer type (private, Medicare, or Uniform, and None or Medicaid), receipt of chemotherapy, and receipt of radiation therapy*.

Compared with patients without metastasis, patients with metastatic breast cancer are more likely to have overtly altered inflammatory states and tumor responses (35, 36) and shorter survival largely attributed to breast cancer. We, thus, analyzed the relationship between NLR and all-cause mortality, after excluding stage IV breast cancer patients (Table 3). The unadjusted HRs associated with high NLR were 2.43 (95% CI: 1.36–4.35) in all patients and 2.78 (95% CI: 1.16–6.64) and 2.28 (95% CI: 1.03–5.03) among Black and White patients, respectively. The high NLR remained a significant independent prognostic factor for overall survival among non-metastatic breast cancer patients after adjustment for the clinical factors of age, stage, grade, and combined ER/PR receptor status (HR = 2.12, 95% CI: 1.05–4.29) and additional adjustment for the patient factors of BMI and smoking history and the treatment factors of payer category, chemotherapy regimen, and radiation therapy regimen (HR = 2.27, 95% CI: 1.02–5.08).

Out of 122 patients who died during the study follow-up, 54 (10 in high NLR group and 44 in low NLR group) deaths were determined attributable to breast cancer. High NLR was significantly associated with breast cancer-specific mortality (unadjusted HR = 2.16, 95 CI: 1.09–4.30) but the association no longer remained after adjustment for clinical factors (adjusted HR = 1.78, 95% CI: 0.71–4.42) (Table 5). Race and tumor metastasis status did not influence the relationship between the NLR and breast cancer-specific mortality.

**Table 5:** **Breast cancer-specific mortality in relation to high NLR (≥3.7) versus low NLR (<3.7)**.

	Black			White			Combined		
	Deaths	HR	95% CI	Deaths	HR	95% CI	Deaths	HR	95% CI
**All stages**									
Unadjusted	33	2.47	(0.95, 6.39)	21	2.15	(0.79, 5.86)	54	2.16	(1.09, 4.3)
Adjustment model 1	24	1.08	(0.30, 3.94)	10	1.41	(0.25, 7.96)	34	1.78	(0.71, 4.42)
Adjustment model 2	22	0.57	(0.12, 2.73)	8	4.74	(0.23, 99.30)	30	1.27	(0.46, 3.45)
Adjustment model 3	22	0.59	(0.10, 3.45)	8	^a^	^a^	30	1.46	(0.46, 4.62)
**Excluding stage IV**									
Unadjusted	17	3.31	(0.95, 11.60)	6	1.51	(0.18, 12.90)	23	2.22	(0.75, 6.53)
Adjustment model 1	16	2.73	(0.75, 10.00)	3	^a^	^a^	19	2.71	(0.86, 8.56)
Adjustment model 2	14	1.40	(0.26, 7.51)	2	^a^	^a^	16	1.52	(0.40, 5.85)
Adjustment model 3	14	1.06	(0.16, 7.16)	2	^a^	^a^	16	1.69	(0.38, 7.55)

*^a^Model did not converge. Adjustment model 1 controlled for stage (0/I, II, III, and IV), grade (I, II, and III/IV), hormone receptor status (ER+ or PR+, ER−, and PR−), and age (continuous). In addition to the variables in adjustment model 1, adjustment model 2 controlled for smoking (former and current versus never) and BMI (<25, 25–2.9, 30–34.9, and 35+). In addition to the variables in adjustment model 2, adjustment model 3 controlled for payer type (private, Medicare, or Uniform, and None or Medicaid), receipt of chemotherapy, and receipt of radiation therapy*.

## Discussion

In a cohort of 461 Black and White patients with breast cancer, elevated pretreatment NLR, defined as 3.7 or higher, was not significantly associated with all-cause or breast cancer-specific mortality after considering patient and clinical factors. By contrast, elevation in pretreatment NLR was an independent predictor of all-cause mortality among breast cancer patients without metastasis at the time of diagnosis.

Some previous studies have reported that NLR is associated with breast cancer-specific mortality. Forget et al. found decreased recurrence-free survival in breast cancer patients with elevated pretreatment NLR, but multivariate analyses were not conducted in that study due to small sample size (15). More recently, Krenn-Pilko et al. reported that NLR >3 is an independent predictor of poor disease-free survival but not of overall survival in 762 European female breast cancer patients (37). Pretreatment NLR ≥2.5 was independently associated with breast cancer-specific mortality in two studies of Asian patients with stage I–III breast cancer (13, 14). One of the studies also showed that elevated NLR was associated with worse disease-specific survival particularly among patients with the luminal A subtype breast cancer (13). On the other hand, a pretreatment NLR ≥2.5 was not associated with the 21-gene recurrence score in a population of 242 ER+ breast cancer patients (38). A significant association of pretreatment NLR with both overall and disease-free survival has been reported in studies based on triple-negative breast cancer patients (19, 39). In a population of 187 HER2-positive breast cancer patients treated with adjuvant trastuzumab in medical centers in Turkey, Ulas et al. reported that patients with a pretreatment NLR >2.38 had shorter disease-free survival, but their findings were non-significant (*p* = 0.45) (40).

Several studies also point to the association of pretreatment NLR with overall survival in breast cancer patients. A meta-analysis of five studies published in 2014 has summarized that elevated pretreatment NLR, with the cutoff values ranged between 2.0 and 4.0, was associated with a significant increase in all-cause mortality (HR = 2.3, 95% CI: 1.1–4.8) but to a lesser extent with disease-free survival (HR = 1.4, 95% CI: 0.9–2.1) (17). Subgroup analyses also indicated that the significant association between pretreatment NLR and prognostic utility of NLR is more evident among White versus Asian patients (17). Consistent with this notion, a recent large study based on patients of different Asian ethnic groups reported a significant, but modest, association between high pretreatment NLR and overall death among breast cancer patients (18). However, to the best of our knowledge, there has been no research that included adequate number of Black patients.

Black individuals have lower WBC and absolute neutrophil counts compared with White individuals (32) primarily due to genetic deletion of the Duffy antigen receptor for chemokines that has been hypothesized to affect the number of circulating neutrophils (41, 42). Consistent with this understanding, Black patients in our dataset had lower NLR values than White patients, even after stratifying patients by tumor stage. This trend led us to determine different optimal NLR cutoff points for Black and White patients separately. Reflecting an overall lower NLR value in Black patients, a slightly lower NLR cutoff was selected for Black patients compared with White patients, but the difference was not large enough to warrant race-specific cutoff points. We also found that the association between pretreatment NLR value and tumor stage was less evident among Black patients. However, the association between NLR and all-cause and breast cancer-specific mortality remained similar with comparable estimates using either race-specific or a unified cutoff point value, and there was no evidence suggesting racial heterogeneity of the prognostic utility of NLR.

Inflammation and host immune response can be a critical factor for breast cancer prognosis by altering the tumor microenvironment and therapeutic efficacy (43). Peripheral immune parameters, such as NLR studied in the present study, are a plausible and practically attractive marker of systemic inflammatory responses (44) and may reflect tumor-specific immune response. In a recent study of non-small cell lung cancer patients, the high NLR was correlated with TILs and independently associated with the prognosis (45). However, NLR largely represents a non-specific indicator of an immune state (46). For example, an elevated NLR has been linked to worse outcomes in various diseases across several different cancer types and those outside of cancer, including coronary artery disease and heart failure (47–49). In a large cohort study that included both patients with and without cancer, high neutrophil and low lymphocyte counts were associated with an increase in all-cause mortality and cardiovascular mortality as well as cancer-specific mortality (48). It is known that women, especially elderly women, diagnosed with early stage breast cancers are more likely to die because of cardiovascular diseases than from breast cancer (50). We were unable to estimate cardiovascular disease-specific mortality or its contribution to the observed association between NLR and all-cause mortality in our cohort. However, these findings, including our study, emphasize the pervasive effect that inflammation has on several aspects of health in several different organ systems and processes. It should be also noted that although this study focused on NLR, the prognostic implication of inflammation could not be fully captured, using this single marker of inflammation. However, combined with other markers of local and systemic inflammation, NLR, a low-cost, reliable marker of inflammation may have potential utility in predicting short- and long-term prognosis and risk stratification for anti-inflammatory or immune therapy for cancer patients in the future.

Several strengths and limitations exist within the study. In terms of strengths, this study is one of the first to determine the association of pretreatment NLR to both all-cause and breast cancer-specific mortality in a group of Black and White patients. Nearly, equal numbers of Black and White patients were included, allowing us to explore a meaningful cutoff point in both racial groups. We used a cutoff point determination method for survival analysis to select a cutoff point that maximizes the relation with the HRs and SEs in Black and White patients (33, 34). Because the distribution of NLR varies in the two racial groups and the biological meaning in terms of breast cancer prognosis is unknown, we believe this method was well suited to the nature of our data and the specific purpose of our study to examine the association between NLR and prognosis in Black and White patients. However, the prognostic value of our chosen cutoff point can be only determined after validation in independent datasets.

In terms of weaknesses, our study relied on medical records to identify breast cancer-specific death based on *a priori* criteria. Our data had comparable numbers of breast cancer-specific deaths by stage to those reported in a previous national death registry-based study (51), but some unquantifiable level of classification bias is unavoidable. The nature of a retrospective chart review created additional limitations to our study. First, while we began with a cohort of 461 patients, we lost several patients in subsequent models adjusting for potential confounding factors due to incomplete patient records. Thus, while the adjustment models became subsequently more specific in their analysis, they may have lost statistical significance due to decreasing numbers of patients included in the model. Second, we lacked patient data on cardiometabolic health status and cardiovascular health risk factors. As NLR has been previously shown to be associated with increased prevalence of coronary artery disease and cardiovascular mortality (48, 49, 52–55), the relationship between NLR and cardiometabolic status in our patient cohort could have further weakened the specificity of NLR for breast cancer-specific mortality. Finally, the present study conducted subgroup analyses by race and metastasis status at diagnosis. Although the analyses were planned and guided by biological plausibility, the resulting increased number of testing will raise the probability of false positive findings.

The results of the present retrospective cohort of 461 Black and White breast cancer patients showed that elevated NLR defined as an NLR of 3.7 or higher is an independent predictor of all-cause mortality in non-metastatic breast cancer patients. Further studies, preferably in a patient population with available cardiovascular health data, are needed to clarify the appropriate clinical context to use NLR as a prognostic factor.

## Author Contributions

JR, JC, JK, S-CT, and SK designed the study presented in this manuscript. JR and SK collected data from the electronic health records for this study. JC and JK performed the statistical analyses for this study. JR, JC, and SK wrote the manuscript and created the tables and figures with input from JK and S-CT.

## Conflict of Interest Statement

The authors declare that this research was conducted in the absence of any commercial or financial relationships that could be construed as a potential conflict of interest.

## Abbreviations

NLR, neutrophil/lymphocyte ratio (the ratio of peripheral blood neutrophils to peripheral blood lymphocytes, obtained from the differential counts of a complete blood count performed on venous blood).
